# Knowledge, attitudes and practices towards community-acquired pneumonia and COVID-19 among general population: a cross-sectional study

**DOI:** 10.1186/s13756-023-01361-6

**Published:** 2024-01-17

**Authors:** Er Hong, Jia Mao, Zhicheng Ke, Wei Tao

**Affiliations:** 1 Department of Respiratory, Ningbo Municipal Hospital of Traditional Chinese Medicine, Affiliated to Zhejiang Chinese Medical University, Ningbo, 315010 China; 2 Department of Radiology, Ningbo Municipal Hospital of Traditional Chinese Medicine, Affiliated to Zhejiang Chinese Medical University, Ningbo, 315010 China

**Keywords:** Knowledge attitudes practices, Community-acquired pneumonia, COVID-19

## Abstract

**Background:**

This study aimed to assess the knowledge, attitudes, and practices (KAP) of the general population to community-acquired pneumonia (CAP) and COVID-19.

**Methods:**

A cross-sectional study was conducted between September 2022 and February 2023, involving the general population from Ningbo Municipal Hospital of Traditional Chinese Medicine with a self-developed questionnaire.

**Results:**

A total of 637 valid questionnaires were collected, with the majority of participants being female (62.48%). The mean score for knowledge, attitudes, and practices were 7.60 ± 2.39 (possible range: 0–12), 43.20 ± 4.57 (possible range: 11–55), and 34.57 ± 4.95 (possible range: 10–50), respectively. Multivariate logistic regression analysis indicated that master’s degree or above (OR = 6.04, 95% CI: 1.80-20.31, P = 0.004) and occupation in business or service careers (OR = 0.28, 95% CI: 0.17–0.48, P < 0.001) were independent associated with knowledge. The knowledge (OR = 1.32, 95%CI: 1.20–1.44, P < 0.001) and female gender (OR = 1.48, 95%CI: 1.03–2.14, P = 0.036) were independently associated with positive attitudes. Attitudes (OR = 1.34, 95%CI: 1.26–1.43, P < 0.001) and a monthly household income greater than 20,000 RMB (OR = 0.31, 95%CI: 0.15–0.64, P = 0.001) were independent associated with practices. Pearson correlation analysis revealed that knowledge positively correlated with attitude scores (r = 0.348, P < 0.001) and practice scores (r = 0.259, P < 0.001), and attitude and practice scores were also positively correlated (r = 0.563, P < 0.001). Structural equation modeling showed that knowledge predicted attitudes (β = 0.67, P < 0.001) and practices (β = 0.17, P = 0.017), while attitudes predicted practices (β = 0.58, P < 0.001).

**Conclusion:**

General population had moderate knowledge, positive attitudes and average practices towards CAP and COVID-19.

**Supplementary Information:**

The online version contains supplementary material available at 10.1186/s13756-023-01361-6.

## Background

Community-acquired pneumonia (CAP) is a term used to describe pneumonia that is acquired outside of healthcare facilities. It is predominantly caused by bacterial, viral, or fungal infections and is a leading cause of hospitalization, mortality, and substantial healthcare expenses [1–3]. CAP affects a significant number of individuals worldwide, with estimates suggesting up to 450 million cases annually. Developing countries, including China, bear a substantial burden of emerging CAP cases, with approximately 21 million people affected in China alone. These statistics underscore the global impact of CAP and the urgent need for effective prevention, diagnosis, and management strategies, particularly in resource-limited settings [4].

COVID-19, caused by the novel coronavirus SARS-CoV-2, has emerged as a significant viral cause of CAP. This highly contagious respiratory illness has rapidly spread worldwide, leading to a global pandemic since late 2019. COVID-19 primarily affects the respiratory system and presents with symptoms such as fever, dry cough, and shortness of breath, commonly observed in affected individuals. The global impact of COVID-19 has necessitated comprehensive efforts to mitigate transmission, manage symptoms, and develop effective prevention and control strategies [5]. It is important to note that approximately 5% of COVID-19 patients may experience severe respiratory distress, multiorgan dysfunction, and even death, especially among vulnerable populations such as the elderly and individuals with underlying medical conditions like hypertension and diabetes [6–8]. COVID-19 has had a significant global impact, with over 272 million confirmed cases and more than 5.3 million deaths reported worldwide. These numbers continue to rise as the pandemic persists [9]. Therefore, it is crucial to implement population-focused approaches that promote preventive measures like vaccination, good hygiene practices, early diagnosis, and timely treatment in order to effectively manage COVID-19.

Knowledge, attitudes, and practices (KAP) assessments are widely used in public health research to understand people’s understanding, attitudes, and behaviors related to specific health issues [10, 11]. Knowledge, which includes the comprehension of facts, information, and principles, plays a critical role in the successful prevention and control of respiratory infections. Attitudes refer to an individual’s personal feelings, beliefs, and values that influence their behavior, while practices refers to the actual behaviors exhibited in response to an illness. In the context of respiratory illnesses such as CAP and COVID-19, KAP assessments provide valuable insights into the community’s perceptions and responses to these diseases. Despite numerous KAP studies towards COVID-19, no consistent results have been reached to date, and there remains a research gap about comprehensive and updated evaluation of KAP towards COVID-19 in China [12, 13]. Additionally, there is a lack of available studies that explore the public’s KAP of COVID-19 specifically in the context of CAP. This knowledge gap hampers the development of effective prevention and control strategies for CAP.

The objective of this study was to assess the knowledge, attitudes, and practices (KAP) status of the general population in China towards CAP and COVID-19. The aim was to identify areas that require improvement in public health interventions.

## Methods

### Study design and participants

This cross-sectional study was conducted at Ningbo Municipal Hospital of Traditional Chinese Medicine from September 2022 to February 2023. The participants were recruited from the general population visiting the hospital. The inclusion criteria for participants were as follows: (1) having full behavioral capacity; (2) being aged between 18 and 75 years. The exclusion criteria were: (1) participants who have mental illness impairing proper communication; (2) those who have severe memory impairment affecting their ability to recall past events. The study was approved by the Medical Ethics Committee of Ningbo Municipal Hospital of Traditional Chinese Medicine (No. 202200501), and informed consent was obtained from all study participants.

### Procedures

The questionnaire design was based on *Chinese Guidelines for the Diagnosis and Treatment of Adults with Community-acquired Pneumonia (2016 Edition)* [14], *Diagnosis and Treatment of adults with Community-acquired Pneumonia* [15], *Diagnosis and Treatment Protocol for Novel Coronavirus Pneumonia (Trial Version 7)* [16] and related literatures [17–19]. A pilot study was conducted with 30 participants. The results demonstrated that the questionnaire exhibited high internal consistency, as indicated by a Cronbach’s α value of 0.925.

The final version of the questionnaire was in Chinese and contained four dimensions: the demographic information included gender, age, marital status, highest education level, monthly household income, occupation, underlying lung diseases, and previous history of COVID-19. The knowledge dimension of the questionnaire consisted of 12 questions Participants received one point for each correct answer and zero points for incorrect or unclear responses, from 0 to 12 points. The attitudes dimension of the questionnaire comprised 11 questions, and participants were asked to rate their responses on a five-point Likert scale. The scale ranged from “very positive” (5) to “very negative” (1). The scoring range for this dimension was from 11 to 55 points. The practices dimension of the questionnaire consisted of 12 questions. Ten of these questions utilized a five-point Likert scale, ranging from “always” (5 points) to “never” (1 point). The scoring range for these nine questions was from 10 to 50 points. The remaining two practice questions were open-ended and were not assigned scores.

The questionnaires were administered using both paper and online forms. Paper form questionnaires were randomly distributed in the hospital and completed voluntarily by general populations seeking medical treatment. Online electronic questionnaires were distributed through a specialized online survey platform called “Questionnaire Star” (Changsha Ranxing Information Technology Co., Ltd.). Participants completed the online questionnaires by scanning the QR code provided to them when they visited the hospital for medical treatment. The obtained results were compiled and summarized using Excel. The research team meticulously reviewed all questionnaires for completeness, consistency, and validity.

### Statistical analysis

Stata 17.0 (Stata Corporation, College Station, TX, USA) was used for this study. Continuous variables were presented as mean and standard deviation (SD), while categorical data were expressed as n (%). For continuous variables with a normal distribution, Student’s t-test was utilized to compare two groups, while the Mann-Whitney test was employed for continuous variables without a normal distribution. ANOVA and Kruskal-Wallis analysis of variance were utilized for the comparison of continuous variables with and without a normal distribution and homogeneity of variance among three or more groups, respectively. Pearson’s correlation analysis was applied to analyze the correlations between knowledge, attitude, and practice scores. Multivariate logistic regression analysis was performed to examine the relationship between demographic information, knowledge, attitudes, and practices, using the 70% of the KAP score distribution as the cut-off point. Structural Equation Modeling (SEM) was employed to test the following hypotheses: knowledge affects attitudes, knowledge affects practices, knowledge also influences practices through attitudes, and attitudes directly influences practices. The independent risk factors from demographic information variables were utilized as hypotheses applied to the SEM model to determine their impact on knowledge, attitudes, and practices. Tests for collinearity were conducted using a variance inflation factor (VIF). VIF ≤ 10 of all variables and KAP scores were included in the final model. A two-sided p < 0.05 was considered statistically significant.

## Results

A total of 637 questionnaires were included for analysis (Table 1). The majority of participants fell within the age range of 31–50 years, comprising 62.64% of the sample. Females accounted for the majority of the population at 62.48%, and 74.41% of the participants were married. Furthermore, a large proportion of the participants (81.47%) had obtained a bachelor’s degree or higher education, with 63.90% reporting a monthly income between 5000 and 20,000 RMB. In terms of occupation, a significant percentage worked as professionals or technical personnel (34.69%), followed by those in business or service sectors (20.41%). Moreover, the majority of respondents (88.07%) reported no underlying lung diseases, such as chronic obstructive pulmonary disease (COPD) or chronic bronchitis. However, a substantial proportion of the sample (77.39%) reported having been infected with SARS-CoV-2.

**Table 1:** Demographic information and KAP scores

Variables	N (%)	Knowledge		Attitudes		Practices	
		Mean ± SD	*P*	Mean ± SD	*P*	Mean ± SD	*P*
**Total**	637	7.60 ± 2.39		43.20 ± 4.57		34.57 ± 4.95	
**Gender**			0.463		0.040		0.508
Male	239(37.52)	7.51 ± 2.59		42.72 ± 4.68		34.40 ± 4.90	
Female	398(62.48)	7.66 ± 2.27		43.49 ± 4.49		34.67 ± 4.98	
**Age**			< 0.001		0.011		0.208
≤ 30	152(23.86)	8.16 ± 2.45		43.64 ± 4.68		35.01 ± 5.40	
31–40	227(35.64)	7.79 ± 2.24		43.72 ± 4.36		34.80 ± 5.10	
41–50	172(27.00)	7.35 ± 2.36		42.63 ± 4.48		33.94 ± 4.40	
≥ 51	86(13.50)	6.63 ± 2.40		42.21 ± 4.87		34.43 ± 4.69	
**Marital status**			0.002		0.260		0.095
Unmarried/divorced/widowed	163(25.59)	8.10 ± 2.46		43.55 ± 4.65		35.12 ± 5.28	
Married	474(74.41)	7.43 ± 2.34		43.08 ± 4.54		34.37 ± 4.82	
**Education**			< 0.001		0.005		0.009
Junior high school and below	33(5.18)	5.48 ± 2.51		40.91 ± 4.71		32.45 ± 6.21	
Senior high school	85(13.34)	6.48 ± 2.17		42.42 ± 4.71		33.56 ± 4.54	
Bachelor	464(72.84)	7.79 ± 2.27		43.48 ± 4.34		34.89 ± 4.87	
Master and above	55(8.63)	9.05 ± 2.18		43.42 ± 5.64		34.60 ± 5.01	
**Monthly household income**			0.009		0.092		0.426
< 5000 yuan	92(14.44)	7.08 ± 2.49		42.27 ± 5.08		34.83 ± 5.54	
5000–10,000 yuan	207(32.50)	7.41 ± 2.63		43.53 ± 4.35		34.78 ± 5.44	
10,000–20,000 yuan	200(31.40)	7.74 ± 2.23		43.51 ± 4.49		34.64 ± 4.48	
> 20,000 yuan	138(21.66)	8.06 ± 2.07		42.89 ± 4.59		33.96 ± 4.37	
**Occupation**			< 0.001		< 0.001		0.009
Professional and technical personnel (teachers, doctors, engineers and technicians, writers and other professionals)	221(34.69)	8.54 ± 2.18		44.22 ± 4.27		35.39 ± 4.65	
Business, service personnel	130(20.41)	6.88 ± 2.18		42.91 ± 4.34		34.15 ± 5.09	
Others	286(44.90)	7.21 ± 2.43		42.56 ± 4.76		34.12 ± 5.04	
**Any underlying lung diseases (chronic obstructive pulmonary disease (COPD)/chronic bronchitis)**			0.002		0.025		0.239
Yes	42(6.59)	6.69 ± 2.34		42.38 ± 5.10		35.02 ± 5.92	
No	561(88.07)	7.73 ± 2.38		43.37 ± 4.50		34.61 ± 4.86	
Unclear	34(5.34)	6.74 ± 2.31		41.41 ± 4.71		33.24 ± 5.09	
**Having been infected with SARS-CoV-2**			0.013		0.106		0.001
Yes	493(77.39)	7.59 ± 2.43		43.11 ± 4.55		34.47 ± 4.92	
No	118(18.52)	7.94 ± 2.10		43.86 ± 4.50		35.60 ± 4.74	
Unclear	26(4.08)	6.42 ± 2.56		42.00 ± 5.04		31.69 ± 5.21	

The participants achieved an average knowledge score of 7.60 ± 2.39 (possible range: 0–12) (Table 1). The knowledge scores were significantly different by age, marital status, educational attainment, monthly household income, occupation type, presence of lung diseases, and SARS-CoV-2 infection status (all P < 0.05). The knowledge section displayed a range of correct response rates, varying from 41.13 to 92.78%. Specifically, 41.13% of participants correctly identified traditional Chinese medicine, such as Lianhua Qingwen capsules, as being included in empiric treatment for community-acquired pneumonia (item 11, Table 2). However, nearly all respondents (92.78%) recognized that fever, dry cough, fatigue, and other symptoms such as nasal congestion, runny nose, sore throat, loss of smell or taste, and muscle pain were the primary clinical manifestations of COVID-19 (item 7, Table 2).

**Table 2:** Distribution of knowledge section

Knowledge Items	Correct Rate N (%)
1. Infection outside the hospital with no symptoms, but the onset in the hospital during the incubation period is also a community-acquired pneumonia.	371(58.24)
2. The probability of community-acquired pneumonia in immunodeficiency patients is the same as that of healthy controls.	339(53.22)
3. Chest pain or chest discomfort is also the common clinical manifestation of community-acquired pneumonia.	376(59.03)
4. The SARS-CoV-2 is contagious during the incubation period.	569(89.32)
5. Healthy individuals can avoid infection from SARS-CoV-2-contaminated objects as long as they hold their breath.	549(86.19)
6. The COVID-19 mainly damages the lungs and has little impact on other organs.	500(78.49)
7. The main clinical manifestations of COVID-19 are fever, dry cough, fatigue, and some patients will suffer from nasal congestion, runny nose, sore throat, loss of smell/taste, muscle pain, diarrhea, etc.	591(92.78)
8. Most children have relatively mild symptoms after being infected with SARS-CoV-2, and some only show digestive tract reactions such as vomiting and diarrhea.	450(70.64)
9. In the case of ineffective community empiric treatment, the identification of community-acquired pneumonia pathogens can be realized by X-ray.	453(71.11)
10. The nucleic acid detection of COVID-19 was based on polymerase chain reaction.	333(52.28)
11. Empiric treatment of community-acquired pneumonia included traditional Chinese medicine such as Lianhua Qingwen capsules.	262(41.13)
12. If patients with COVID-19 have gastrointestinal discomfort during the observation period, these patients can try to use Huo Xiang Zhengqi capsules of TCM therapy.	361(56.67)

The participants had a mean attitude score of 43.20 ± 4.57 (possible range: 11–55). The attitude scores were significantly different by gender, age, education level, occupation type, and lung disease status (all P < 0.05) (Table 1). Additionally, the proportion of participants who answered “Strongly agree” or “Agree” in the attitudes section ranged from 73.2 to 96.8%. Specifically, 96.8% of participants recognized the importance of paying attention to vulnerable populations, such as the elderly and children, in the prevention of community-acquired pneumonia (item 3, Table 3). On the other hand, only 73.2% of respondents expressed agreement with the effectiveness of vaccination in preventing community-acquired pneumonia and COVID-19 (item 11, Table 3).

**Table 3:** Distribution of attitudes section

Attitudes Items	Strongly agree	Agree	Neutral	Disagree	Strongly disagree
1. Are you willing to actively learn about community-acquired pneumonia?	303 (47.6)	226 (35.5)	99 (15.5)	8 (1.3)	1 (0.2)
2. Are you willing to actively learn about COVID-19?	327 (51.3)	230 (36.1)	72 (11.3)	8 (1.3)	0 (0.0)
3. Do you think it is very important to pay attention to vulnerable populations such as the elderly and children with regard to community-acquired pneumonia?	439 (68.9)	178 (27.9)	16 (2.5)	4 (0.6)	0 (0.0)
4. Do you think it is very important to popularize knowledge about community-acquired pneumonia and COVID-19 in the community?	414 (65.0)	201 (31.6)	18 (2.8)	3 (0.5)	1 (0.2)
5. Do you have great trust in doctors’ treatment plans for community-acquired pneumonia and COVID-19?	329 (51.6)	261 (41.0)	32 (5.0)	13 (2.0)	2 (0.3)
6. Do you have confidence that you can strictly follow the doctor’s advice and take medication on time and in the correct dosage?	327 (51.3)	281 (44.1)	21 (3.3)	8 (1.3)	0 (0.0)
7. Do you think that mild adverse reactions to medications are acceptable compared to improvement in disease symptoms?	263 (41.3)	300 (47.1)	32 (5.0)	39 (6.1)	3 (0.5)
8. Are you concerned about serious long-term effects after being infected with community-acquired pneumonia?	189 (29.7)	329 (51.6)	55 (8.6)	59 (9.3)	5 (0.8)
9. Are you concerned about serious long-term effects after being infected with COVID-19?	216 (33.9)	304 (47.7)	53 (8.3)	56 (8.8)	8 (1.3)
10. Do you believe that wearing a mask can prevent the spread of community-acquired pneumonia and COVID-19?	270 (42.4)	271 (42.5)	73 (11.5)	22 (3.5)	1 (0.2)
11. Do you believe that getting vaccinated is effective in preventing community-acquired pneumonia and COVID-19?	224 (35.2)	242 (38.0)	125 (19.6)	33 (5.2)	13 (2.0)

The participants had a mean practice score of 34.57 ± 4.95 (possible range: 10–50). The practice scores were significantly different by education level, occupation type, and SARS-CoV-2 infection status (all P < 0.05) (Table 1). Table 4 further demonstrated that the proportion of participants who reported “Always” or “Often” engaging in recommended practices varied from 13.18 to 90.59%. Notably, a majority of participants (90.59%) expressed a tendency to wear a mask when going out (item 6, Table 4). Conversely, merely 13.18% of the entire sample reported dietary improvements aimed at preventing CAP or COVID-19 infection (item 9, Table 4). Concerning vaccination for CAP and COVID-19, approximately 48.98% received the CAP vaccine, while a striking 95.76% were vaccinated against COVID-19 (item 10–11, Table 4).

**Table 4:** Distribution of practices section

	Definitely/ Always n (%)	Should/Often n (%)	Not sure/Sometimes n (%)	Should not/Rarely n (%)	Definitely not/Never n (%)
1. How often do you proactively learn about community-acquired pneumonia and COVID-19?	145(22.76)	181(28.41)	197(30.93)	98(15.38)	13(2.51)
2. How often do you attend lectures and training on community-acquired pneumonia and COVID-19?	125(19.62)	115(18.05)	180(28.26)	135(21.19)	82(12.87)
3. After seeking medical attention for discomfort, are you able to describe your symptoms clearly to the doctor?	302(47.41)	213(33.44)	84(13.19)	31(4.87)	7(1.10)
4. Can you strictly follow the doctor’s instructions to take medication?	363(56.99)	195(30.61)	67(10.52)	9(1.41)	3(0.47)
5. If you were infected with community-acquired pneumonia or COVID-19, would you experience anxiety, depression, or other emotions?	100(15.70)	114(17.90)	211(33.12)	119(18.68)	93(14.60)
6. Do you wear a mask when you go out?	413(64.84)	164(25.75)	51(8.01)	9(1.41)	0
7. If your family or friends are infected with community-acquired pneumonia or COVID-19, would you pass on correct attitudes and treatment experiences to them?	374(58.71)	184(28.89)	61(9.58)	15(2.35)	3(0.47)
8. Do you exercise regularly to prevent infection with community-acquired pneumonia or COVID-19?	179(28.10)	167(26.22)	170(26.69)	103(16.17)	18(2.83)
9. Do you improve your diet to prevent infection with community-acquired pneumonia or COVID-19?	22(3.45)	62(9.73)	166(26.06)	62(9.73)	22(3.45)
10. Do you prefer to use traditional Chinese medicine to treat community-acquired pneumonia and COVID-19?	181(28.41)	168(26.37)	191(29.98)	65(10.20)	32(5.02)
	Yes, once	Yes, twice	Yes, three times	Yes, more than three times	No
11. Have you been vaccinated against pneumonia? If yes, how many times have you been vaccinated?	67(10.52)	65(10.20)	134(21.04)	46(7.22)	325(51.02)
12. Have you been vaccinated against COVID-19? If yes, how many times have you been vaccinated?	27(4.24)	122(19.15)	365(57.30)	96(15.07)	27(4.24)

The knowledge score was significantly positively correlated with the attitude scores (r = 0.348, P < 0.001) and practice scores (r = 0.259, P < 0.001). The attitude and practice scores were also significantly positively correlated (r = 0.563, P < 0.001) (Table 5).

**Table 5:** Correlation analysis of knowledge, attitudes and practices dimensions of community-acquired pneumonia and COVID-19 among participants

	Knowledge	Attitudes	Practices
**Knowledge**	1		
**Attitudes**	0.348 (*P* < 0.001)	1	
**Practices**	0.259 (*P* < 0.001)	0.563 (*P* < 0.001)	1

**Fig. 1:**
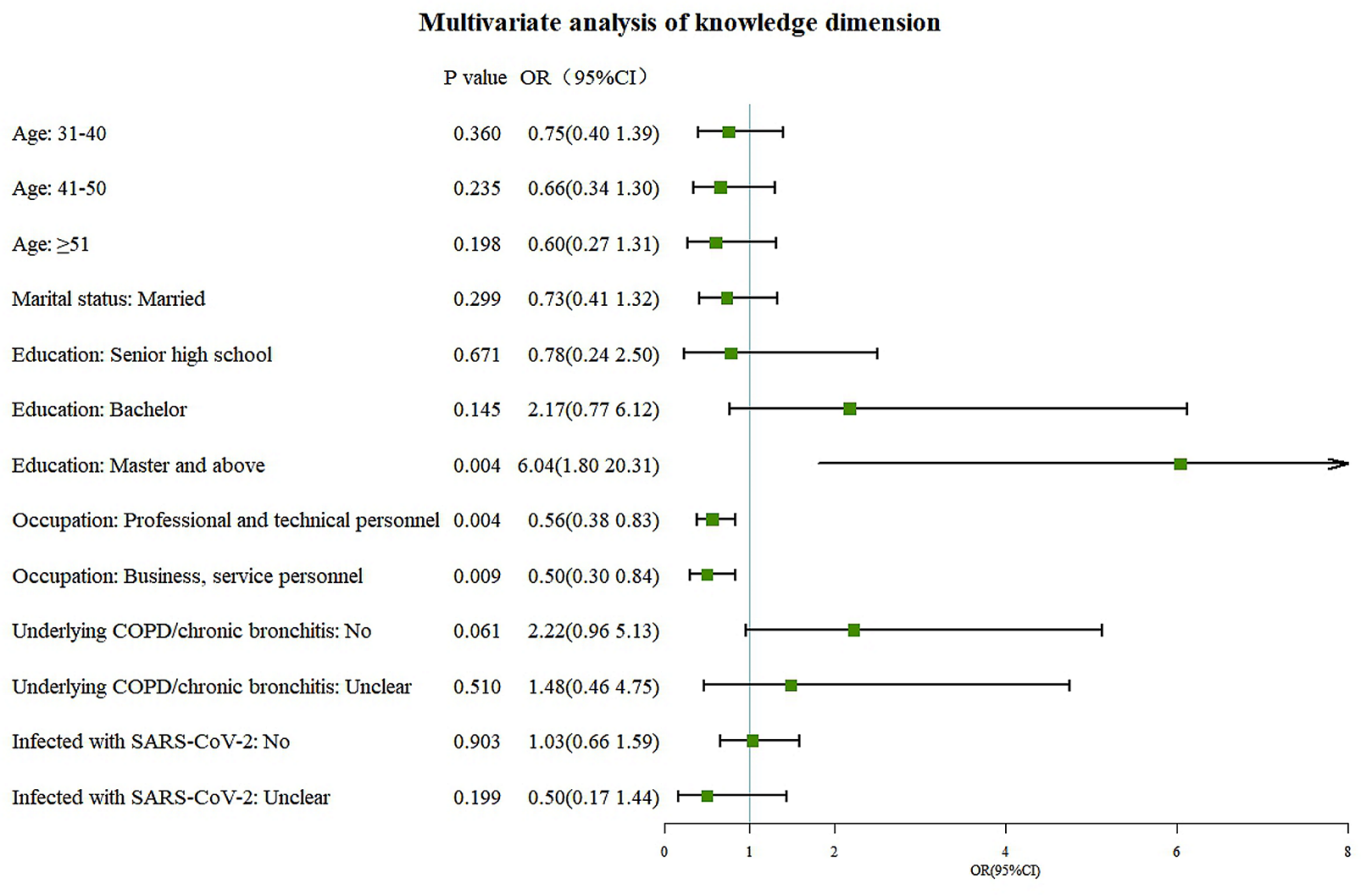
Multivariate logistic regression analysis of knowledge

**Fig. 2:**
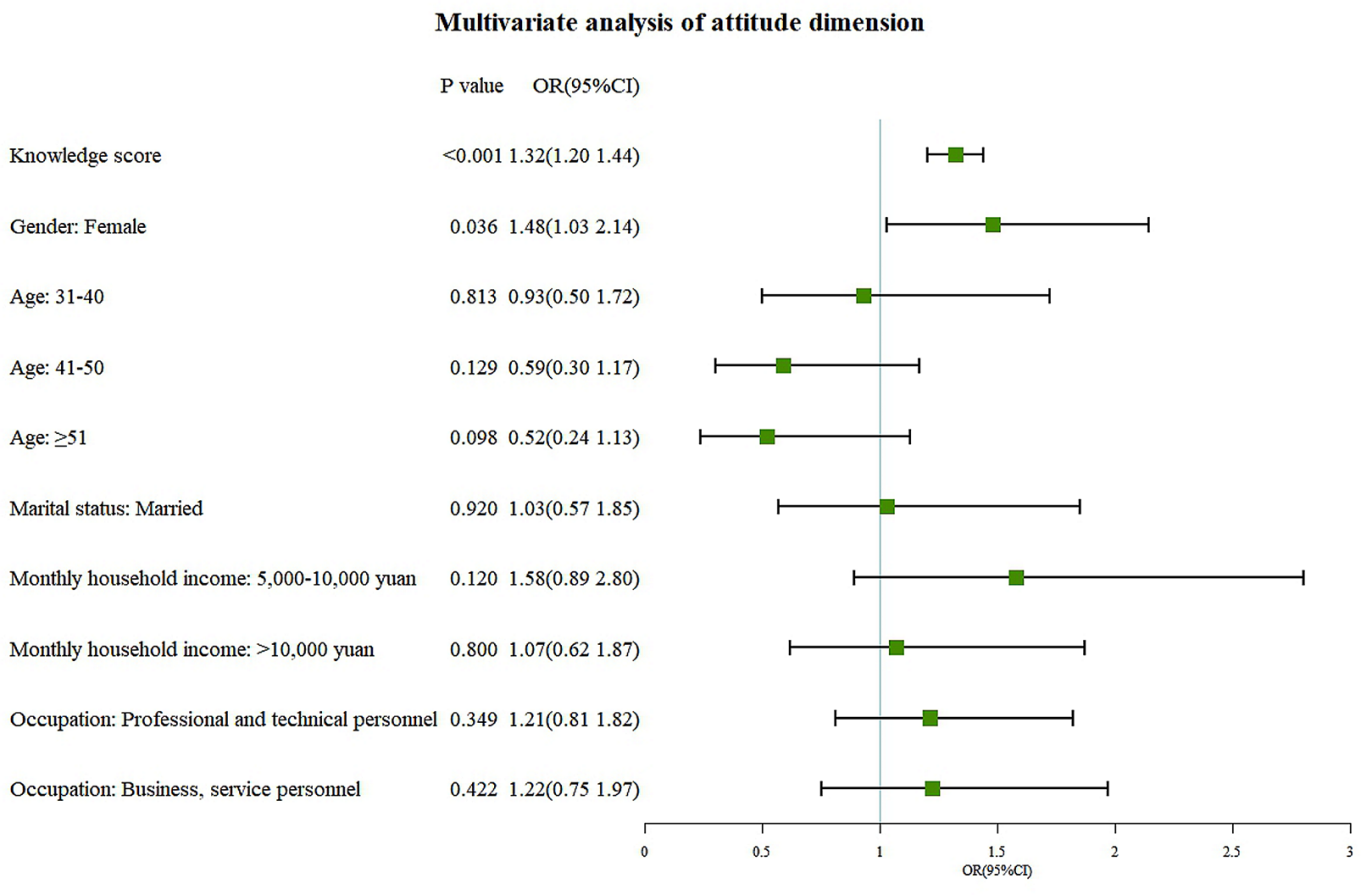
Multivariate logistic regression analysis of attitudes dimension

**Fig. 3:**
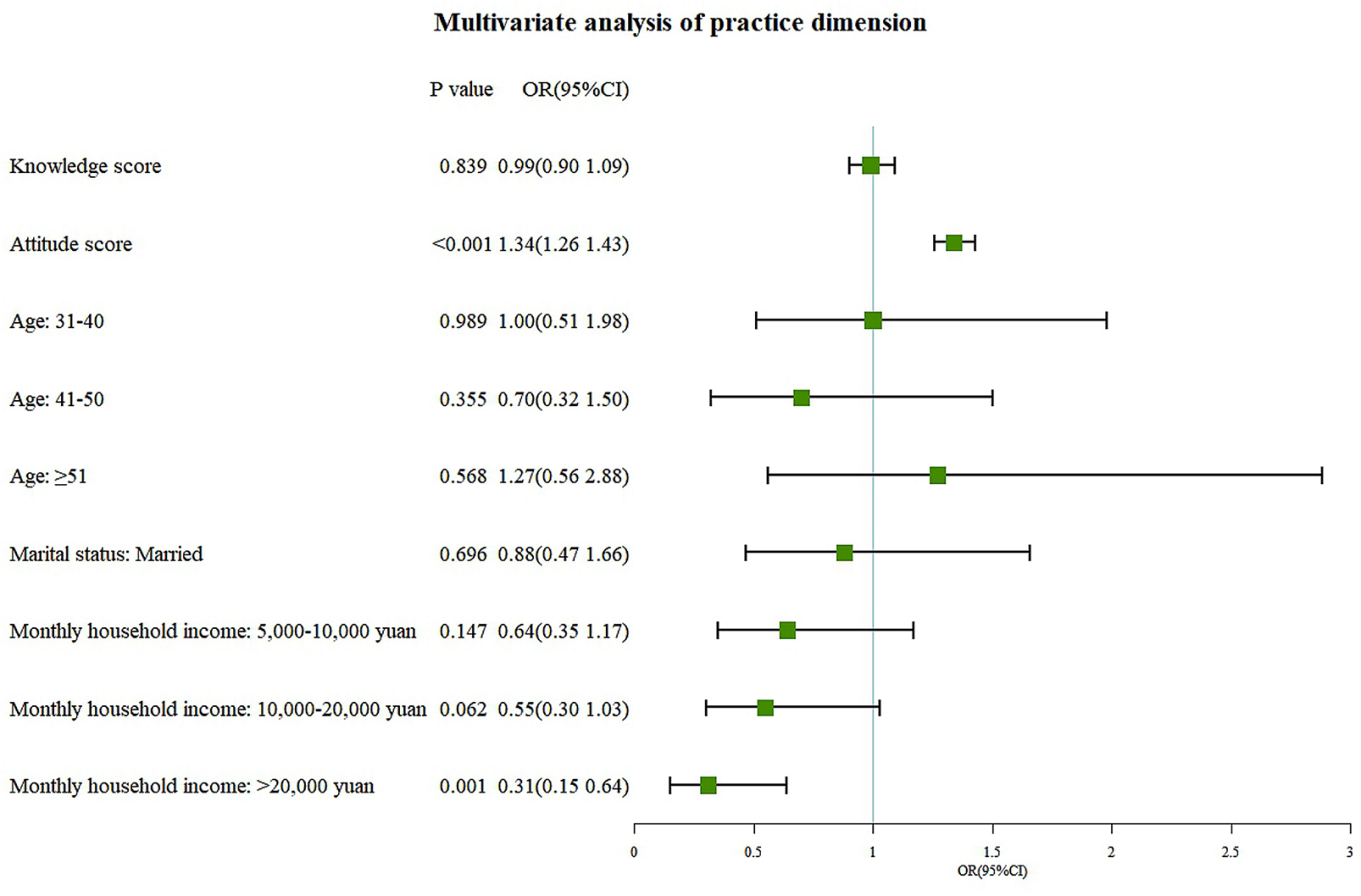
Multivariate logistic regression analysis of practices dimension

Multivariate logistic regression analysis revealed that master’s degree or above (OR = 6.04, 95% CI: 1.80-20.31, P = 0.004) and occupation in business or service careers (OR = 0.28, 95% CI: 0.17–0.48, P < 0.001) were independent associated with knowledge (Fig. 1). Knowledge (OR = 1.32, 95%CI: 1.20–1.44, P < 0.001) and female genders (OR = 1.48, 95%CI: 1.03–2.14, P = 0.036) were independently associated with positive attitudes (Fig. 2). Attitudes (OR = 1.34, 95%CI: 1.26–1.43, P < 0.001) and a monthly household income greater than 20,000 RMB (OR = 0.31, 95%CI: 0.15–0.64, P = 0.001) were independent associated with practices (Fig. 3).

**Fig. 4:**
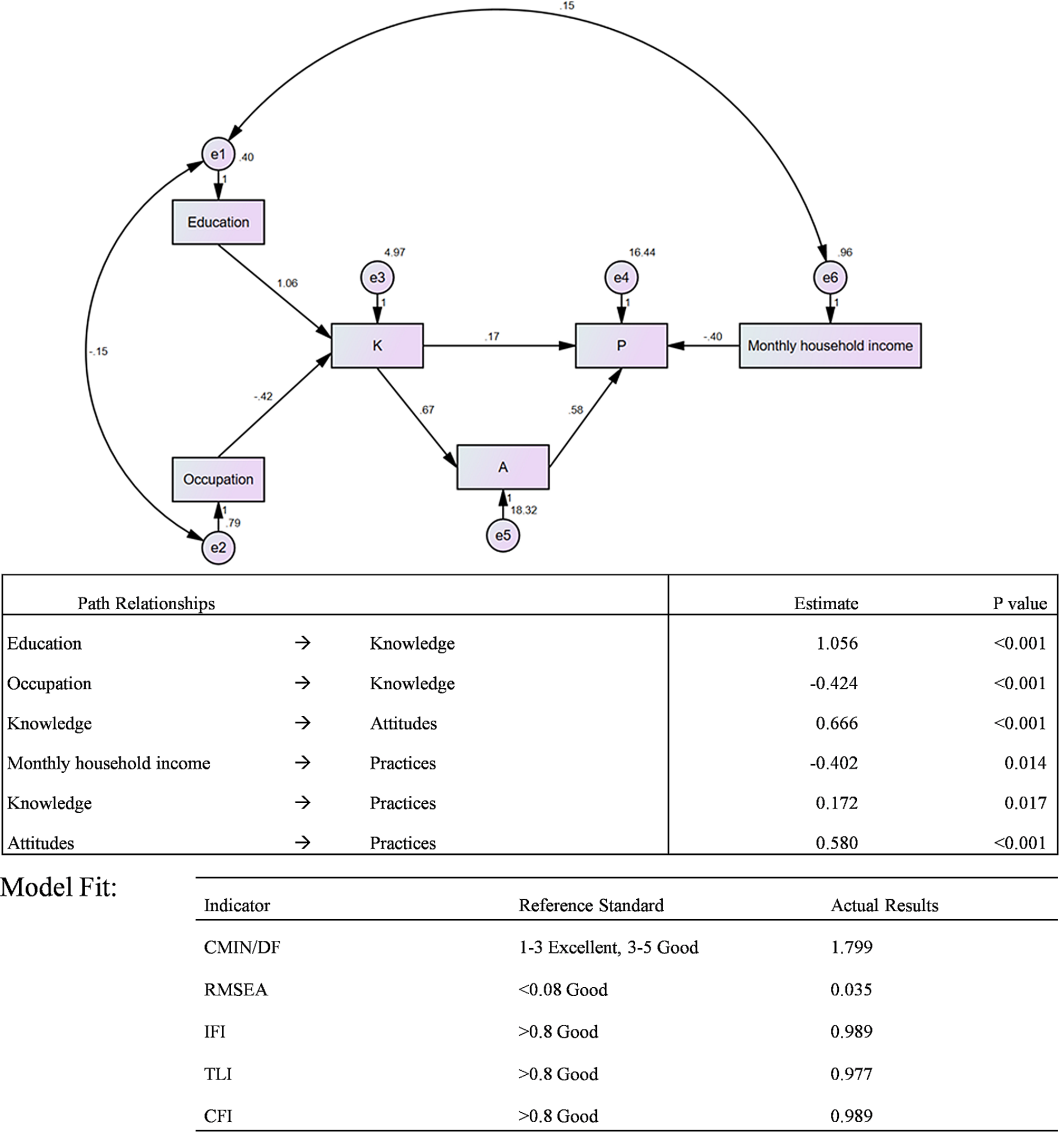
Structure equation model on KAP of community-acquired pneumonia and COVID-19 based on the theory of planned behavior. Standardized path coefficients were presented

A very good fitness of data into the SEM model was found: CMIN/DF = 1.799 (> 1); RMSEA = 0.035 (< 0.08); IFI = 0.989 (> 0.8); TLI = 0.977 (> 0.8) and CFI = 0.989 (> 0.8). The results revealed that knowledge was a potential predictor of attitudes (β = 0.67, P < 0.001) and practices (β = 0.17, P = 0.017), while attitudes seemed can also predict practices (β = 0.58, P < 0.001). Higher education seemed predict good knowledge (β = 1.06, P < 0.001) while occupation of business and service personnel predicted poor knowledge (β=-0.42, P < 0.001). However, higher monthly household income was associated with less practices (β=-0.40, P = 0.014) (Fig. 4).

## Discussion

This study indicate that participants exhibited moderate knowledge, positive attitudes, and medium practices towards CAP and COVID-19. These findings have important implications for public health education and intervention initiatives aimed at enhancing community awareness and participation in preventive measures for respiratory infections.

In the knowledge dimension, a low percentage of participants demonstrated accurate recognition of the inclusion of traditional Chinese medicine, such as Lianhua Qingwen capsules, in empiric treatment for CAP. Published studies have demonstrated the effectiveness of traditional Chinese medicine in combination with Western medicine to enhance clinical outcomes of CAP. The integrative approach has the potential to improve clinical symptoms of CAP, reduce hospitalization duration, and enhance immune responses to ensure better prognosis [20–23]. Efforts should be made to enhance public education and awareness regarding the potential benefits and proper utilization of traditional Chinese medicine in the management of CAP. In addition, it is encouraging to note that the majority of participants accurately recognized the primary symptoms of COVID-19, indicating that public health campaigns and educational initiatives have successfully disseminated information on this topic.

The significant proportion of participants who recognized the importance of vulnerable populations in preventing CAP is a promising finding in the attitudes dimension. The identification of the elderly and children as high-risk groups for CAP is well-supported, considering their compromised immune systems and increased vulnerability to respiratory pathogens [24, 25]. Our findings align with previous studies that emphasize the need for targeted interventions to prevent CAP in vulnerable populations, including vaccination initiatives and educational campaigns. These interventions can effectively reduce the risk of CAP and its associated complications in high-risk groups such as the elderly and children [26, 27]. Therefore, targeting interventions towards these high-risk groups could help decrease the incidence and severity of CAP. Implementing vaccination programs and increasing public awareness about hand hygiene and respiratory etiquette are crucial measures. However, the low percentage of respondents who expressed agreement with the effectiveness of vaccination in preventing CAP and COVID-19 is concerning, considering that vaccination is one of the most effective preventive measures against these diseases [28]. Suboptimal acceptance of vaccination can be attributed to various factors, including misinformation and distrust regarding vaccine safety and effectiveness. Consequently, addressing these concerns through evidence-based educational and communication strategies is crucial, which should include tailored messaging aimed at specific populations.

Majority (90.59%) expressed their commitment to wearing masks when going out, indicating the widespread awareness and compliance with mask-wearing as an effective means to prevent respiratory infections [29]. This high compliance rate could be seen as a positive sign of public awareness during the COVID-19 pandemic. Conversely, only 13.18% of the participants reported making dietary changes to prevent CAP or COVID-19, suggesting that dietary modifications may not be a primary strategy. It could be essential to recognize that dietary factors could influence the immune system’s functioning and overall health [30, 31], however other preventive measures, such as mask-wearing and vaccination, take precedence in the participants’ risk reduction strategies. Regarding vaccination, a clear distinction was observed between the rates of vaccination for CAP and COVID-19. This discrepancy could be attributed to the heightened awareness and urgency associated with the COVID-19 pandemic. This finding highlighted the need for stronger efforts to promote vaccination for other respiratory infections, like CAP, that can also have a significant impact on public health.

Our findings revealed a positive correlation between knowledge and attitudes, which is consistent with previous research [32, 33]. Individuals with higher knowledge and attitude scores towards COVID-19 and CAP demonstrated a greater inclination to actively engage in prevention and treatment. These findings align with previous studies that emphasize the impact of knowledge and attitudes on health-related behaviors. Furthermore, the positive associations observed between education level and knowledge scores, as well as between monthly household income and practice scores, are in line with previous research demonstrating the influence of socioeconomic status on health outcomes and behaviors [34]. The lower knowledge scores observed among participants in business or service careers and other occupations may indicate disparities in access to health information and resources. Additionally, gender differences were identified, with females exhibiting significantly higher attitude scores compared to males. This finding is consistent with prior research suggesting that women are more inclined to engage in health-promoting behaviors and be receptive to health education and messaging. These results underscore the importance of addressing these disparities and tailoring health interventions to different occupational groups and gender-specific needs [35].

However, it is important to acknowledge the limitations of this study, including its small sample size and single-center design, which may have introduced selection bias and limited the generalizability of the results to a broader population. Additionally, the reliance on self-reported responses in the questionnaires may have introduced reporting bias. To address these limitations, future research should consider employing larger sample sizes and conducting multi-center studies to enhance the representativeness of the findings. The use of more comprehensive questionnaires and objective measures could also improve the reliability and validity of the results. Furthermore, controlling for potential confounding variables would provide a more robust analysis of the relationships observed in this study. By addressing these limitations, future studies can provide a more comprehensive understanding of the KAP status of the general population towards CAP and COVID-19.

In summary, the study revealed that the general population displayed a moderate level of knowledge, positive attitudes, and average practices towards CAP and COVID-19. Targeted educational and behavioral interventions are warranted to enhance the prevention and management of both CAP and COVID-19.

### Electronic supplementary material

Below is the link to the electronic supplementary material.

**Supplementary Material 1:** Questionnaire for the Knowledge, attitudes and practices towards community-acquired pneumonia and COVID-19 among general population

## Data Availability

All data generated or analyzed during this study are included in this published article.

## Abbreviations

KAP: Knowledge attitudes and practices
CAP: community-acquired pneumonia
SD: standard deviation
SEM: Structural Equation Modeling
COPD: chronic obstructive pulmonary disease
