# Supplementary material for: Knowledge, attitudes and practices towards community-acquired pneumonia and COVID-19 among general population: a cross-sectional study
Source: Antimicrob Resist Infect Control. 2024 Jan 17;13:6. doi: 10.1186/s13756-023-01361-6 (PMC10795257; doi:10.1186/s13756-023-01361-6)
Supplement: Supplementary file 1 — Supplementary Material 1: Questionnaire for the Knowledge, attitudes and practices towards community-acquired pneumonia and COVID-19 among general population [file 13756_2023_1361_MOESM1_ESM.docx]

**Knowledge, attitudes and practices towards** **community-acquired pneumonia and COVID-19 among general population**

| Part.1 Demographic information | | | | | | | | | | | | | | |  |
| --- | --- | --- | --- | --- | --- | --- | --- | --- | --- | --- | --- | --- | --- | --- | --- |
| 1. Gender： | | a. Male  b. Female | | | | | | |  | | | | | |  |
| 2. Age： | | a. ≤20  b. 21-30  c. 31-40  d. 41-50  e. 51-60  f. ≥60 | | | | | | | | | | | | |  |
| 3. Marital status： | | a. Unmarried  b. Married  c. Divorced  d. Widowed | | | | | | | | | | | | |  |
| 4. Education： | | a. Junior high school and below  b. Senior high school  c. Bachelor  d. Master and above | | | | | | | | | | | | |  |
| 5. Monthly household income： | | a. <2000 yuan  b.2000-5000 yuan  c.5000-10000 yuan  d.10000-20000 yuan  e. >20000 yuan | | | | | | | | | | | | |  |
| 6. Occupation： | | a. Heads of Party and mass organizations of state organs, enterprises and institutions  b. Professional and technical personnel (teachers, doctors, engineers and technicians, writers and other professionals)  c. Clerical and related personnel  d. Business, service personnel  e. Agriculture, forestry, animal husbandry, fishing and water conservancy production personnel  f. Production, transportation equipment operators and related personnel  g. Soldier  h. Others | | | | | | | | | | | | |  |
| 7. Any underlying lung diseases (chronic obstructive pulmonary disease (COPD)/chronic bronchitis)： | | a. Yes  b. No  c. Unclear | | | | | | | | | | | | |  |
| 8. Having been infected with SARS-CoV-2： | | a. Yes  b. No  c. Unclear | | | | | | | | | | | | |  |
| Part.2 Distribution of knowledge section | | | | | | | | | | | | | |  |  |
| 1. Infection outside the hospital with no symptoms, but the onset in the hospital during the incubation period is also a community-acquired pneumonia. | | | | | | | a. Correct | | | | b. Wrong | | c. Unclear | |  |
| 2. The probability of community-acquired pneumonia in immunodeficiency patients is the same as that of healthy controls. | | | | | | | a. Correct | | | | b. Wrong | | c. Unclear | |  |
| 3. Chest pain or chest discomfort is also the common clinical manifestation of community-acquired pneumonia. | | | | | | | a. Correct | | | | b. Wrong | | c. Unclear | |  |
| 4. The SARS-CoV-2 is contagious during the incubation period. | | | | | | | a. Correct | | | | b. Wrong | | c. Unclear | |  |
| 5. Healthy individuals can avoid infection from SARS-CoV-2-contaminated objects as long as they hold their breath. | | | | | | | a. Correct | | | | b. Wrong | | c. Unclear | |  |
| 6. The COVID-19 mainly damages the lungs and has little impact on other organs. | | | | | | | a. Correct | | | | b. Wrong | | c. Unclear | |  |
| 7. The main clinical manifestations of COVID-19 are fever, dry cough, fatigue, and some patients will suffer from nasal congestion, runny nose, sore throat, loss of smell/taste, muscle pain, diarrhea, etc. | | | | | | | a. Correct | | | | b. Wrong | | c. Unclear | |  |
| 8. Most children have relatively mild symptoms after being infected with SARS-CoV-2, and some only show digestive tract reactions such as vomiting and diarrhea. | | | | | | | a. Correct | | | | b. Wrong | | c. Unclear | |  |
| 9. In the case of ineffective community empiric treatment, the identification of community-acquired pneumonia pathogens can be realized by X-ray. | | | | | | | a. Correct | | | | b. Wrong | | c. Unclear | |  |
| 10. The nucleic acid detection of COVID-19 was based on polymerase chain reaction. | | | | | | | a. Correct | | | | b. Wrong | | c. Unclear | |  |
| 11. Empiric treatment of community-acquired pneumonia included traditional Chinese medicine such as Lianhua Qingwen capsules. | | | | | | | a. Correct | | | | b. Wrong | | c. Unclear | |  |
| 12. If patients with COVID-19 have gastrointestinal discomfort during the observation period, these patients can try to use Huo Xiang Zhengqi capsules of TCM therapy. | | | | | | | a. Correct | | | | b. Wrong | | c. Unclear | |  |
| Part.3 Distribution of attitudes section | | | | | | | | | | | | | | | |
| 1. Are you willing to actively learn about community-acquired pneumonia? | a. Strongly agree | | | b. Agree | | c. Neutral | | | | d. Disagree | | e. Strongly disagree | | | |
| 2. Are you willing to actively learn about COVID-19? | a. Strongly agree | | | b. Agree | | c. Neutral | | | | d. Disagree | | e. Strongly disagree | | | |
| 3. Do you think it is very important to pay attention to vulnerable populations such as the elderly and children with regard to community-acquired pneumonia? | a. Strongly agree | | | b. Agree | | c. Neutral | | | | d. Disagree | | e. Strongly disagree | | | |
| 4. Do you think it is very important to popularize knowledge about community-acquired pneumonia and COVID-19 in the community? | a. Strongly agree | | | b. Agree | | c. Neutral | | | | d. Disagree | | e. Strongly disagree | | | |
| 5. Do you have great trust in doctors' treatment plans for community-acquired pneumonia and COVID-19? | a. Strongly agree | | | b. Agree | | c. Neutral | | | | d. Disagree | | e. Strongly disagree | | | |
| 6. Do you have confidence that you can strictly follow the doctor's advice and take medication on time and in the correct dosage? | a. Strongly agree | | | b. Agree | | c. Neutral | | | | d. Disagree | | e. Strongly disagree | | | |
| 7. Do you think that mild adverse reactions to medications are acceptable compared to improvement in disease symptoms? | a. Strongly agree | | | b. Agree | | c. Neutral | | | | d. Disagree | | e. Strongly disagree | | | |
| 8. Are you concerned about serious long-term effects after being infected with community-acquired pneumonia? | a. Strongly agree | | | b. Agree | | c. Neutral | | | | d. Disagree | | e. Strongly disagree | | | |
| 9. Are you concerned about serious long-term effects after being infected with COVID-19? | a. Strongly agree | | | b. Agree | | c. Neutral | | | | d. Disagree | | e. Strongly disagree | | | |
| 10. Do you believe that wearing a mask can prevent the spread of community-acquired pneumonia and COVID-19? | a. Strongly agree | | | b. Agree | | c. Neutral | | | | d. Disagree | | e. Strongly disagree | | | |
| 11. Do you believe that getting vaccinated is effective in preventing community-acquired pneumonia and COVID-19? | a. Strongly agree | | | b. Agree | | c. Neutral | | | | d. Disagree | | e. Strongly disagree | | | |
| Part.4 Distribution of practices section | | | | | | | | | | | | | | |  |
| 1. How often do you proactively learn about community-acquired pneumonia and COVID-19? | | | a. Definitely/Always | | b. Should/Often | | | c. Not sure/Sometimes | | | d. Should  not/Rarely | e. Definitely not/Never | | |  |
| 2. How often do you attend lectures and training on community-acquired pneumonia and COVID-19? | | | a. Definitely/Always | | b. Should/Often | | | c. Not sure/Sometimes | | | d. Should  not/Rarely | e. Definitely not/Never | | |  |
| 3. After seeking medical attention for discomfort, are you able to describe your symptoms clearly to the doctor? | | | a. Definitely/Always | | b. Should/Often | | | c. Not sure/Sometimes | | | d. Should  not/Rarely | e. Definitely not/Never | | |  |
| 4. Can you strictly follow the doctor's instructions to take medication? | | | a. Definitely/Always | | b. Should/Often | | | c. Not sure/Sometimes | | | d. Should  not/Rarely | e. Definitely not/Never | | |  |
| 5. If you were infected with community-acquired pneumonia or COVID-19, would you experience anxiety, depression, or other emotions? | | | a. Definitely/Always | | b. Should/Often | | | c. Not sure/Sometimes | | | d. Should  not/Rarely | e. Definitely not/Never | | |  |
| 6. Do you wear a mask when you go out? | | | a. Definitely/Always | | b. Should/Often | | | c. Not sure/Sometimes | | | d. Should  not/Rarely | e. Definitely not/Never | | |  |
| 7. If your family or friends are infected with community-acquired pneumonia or COVID-19, would you pass on correct attitudes and treatment experiences to them? | | | a. Definitely/Always | | b. Should/Often | | | c. Not sure/Sometimes | | | d. Should  not/Rarely | e. Definitely not/Never | | |  |
| 8. Do you exercise regularly to prevent infection with community-acquired pneumonia or COVID-19? | | | a. Definitely/Always | | b. Should/Often | | | c. Not sure/Sometimes | | | d. Should  not/Rarely | e. Definitely not/Never | | |  |
| 9. Do you improve your diet to prevent infection with community-acquired pneumonia or COVID-19? | | | a. Definitely/Always | | b. Should/Often | | | c. Not sure/Sometimes | | | d. Should  not/Rarely | e. Definitely not/Never | | |  |
| 10. Have you been vaccinated against pneumonia? If yes, how many times have you been vaccinated? | | | a. Yes, once | | b. Yes, twice | | | c. Yes, three times | | | d. Yes, more than three times | e. No | | |  |
| 11. Have you been vaccinated against COVID-19? If yes, how many times have you been vaccinated? | | | a. Yes, once | | b. Yes, twice | | | c. Yes, three times | | | d. Yes, more than three times | e. No | | |  |
| 12. Do you prefer to use traditional Chinese medicine to treat community-acquired pneumonia and COVID-19? | | | a. Definitely/Always | | b. Should/Often | | | c. Not sure/Sometimes | | | d. Should  not/Rarely | e. Definitely not/Never | | |  |
